# Draft Genome Sequence of *Firmicutes* Strain S^0^AB, a Heterotrophic Iron/Sulfur-Oxidizing Extreme Acidophile

**DOI:** 10.1128/mra.00271-22

**Published:** 2022-07-07

**Authors:** Eva Vergara, Eva Pakostova, D. Barrie Johnson, David S. Holmes

**Affiliations:** a Center for Bioinformatics and Genome Biology, Centro Ciencia & Vida, Fundación Ciencia & Vida, Santiago, Chile; b Facultad de Medicina y Ciencia, Universidad San Sebastián, Santiago, Chile; c School of Natural Sciences, Bangor University, Bangor, United Kingdom; d Health and Life Sciences, Coventry University, Coventry, United Kingdom; e Natural History Museum, London, United Kingdom; Montana State University

## Abstract

The draft whole-genome sequence of the extremely acidophilic and novel *Firmicutes* strain S^0^AB is reported. The genome comprises 3.3 Mbp and has a GC content of 43.72%. In total, 3,240 protein-coding genes, 56 tRNA genes, and 11 rRNA genes were predicted.

## ANNOUNCEMENT

The novel Gram-positive, endospore-forming *Firmicutes* strain S^0^AB, which is mesophilic and extremely acidophilic, was isolated from laboratory-grade S^0^ (elemental) sulfur obtained from Peak Trading Company (Nottingham, UK) on a low-pH solid medium containing potassium tetrathionate and ferrous sulfate (1, 2). It requires an organic source of carbon for growth. It can oxidize and reduce iron and catalyzes the dissimilatory oxidation of S^0^ and tetrathionate. S^0^AB was cultured aerobically at 32°C in a liquid basal salt medium containing 20 mM ferrous sulfate and 0.02% (wt/vol) yeast extract, with an initial pH of 1.7 (3). DNA was extracted in two steps, as follows: (i) cells were lysed at 37°C with lysozyme and RNase A, and (ii) proteinase K and SDS were added for incubation at 65°C. DNA was purified using solid-phase reversible immobilization (SPRI) beads (Qiagen) and quantified with the Quant-iT double-stranded DNA (dsDNA) high-sensitivity (HS) kit (Thermo Fisher Scientific). The Illumina library was prepared using the Nextera XT library preparation kit (Illumina) with a Microlab STAR automated liquid-handling system (Hamilton Bonaduz AG, Switzerland) and was sequenced with the high-throughput Illumina NovaSeq 6000 system (producing 907,704 paired-end raw reads of 2 × 250 bp). The library for long-read DNA was prepared with the SQK-RBK004 kit (Oxford Nanopore Technologies [ONT], UK), loaded in a FLO-MIN106 (R.9.4.1) flow cell, and sequenced with a GridION system (ONT) (producing 6,814 raw reads with a mean length of 14,720 bp). Illumina reads were trimmed using Trimmomatic v0.30 with a sliding window quality cutoff of Q15 (4), and quality was assessed using SAMtools (5, 6), BEDTools (7), and BWA-MEM (bio-bwa.sourceforge.net), generating 889,712 raw reads. Hybrid assembly of the reads was performed using Unicycler v0.4.0 (8). Assembly metrics were calculated using QUAST (9). The genome size of S^0^AB is 3,342,730 bp, consisting of 150 contigs (size ≥200 bp; *N*_50_, 247,888 bp) with a GC content of 43.72% and mean coverage of 128×. CheckM (10) analysis showed 97.8% completeness with <3.5% contamination. The genome was annotated using Prokka 1.14.6 (11), which predicted 3,240 protein-coding genes, including genes for 1,519 hypothetical proteins and 1,721 proteins with predicted functional assignments, as well as 56 tRNA genes and 11 rRNA genes. Default parameters were used for all software unless otherwise noted.

The genome contains *sor*, *sdoB*, and *soxB*, which are involved in the dissimilatory oxidation of S^0^ and tetrathionate, and *sqr*, *sat*, and *cysC*, which are involved in the uptake of sulfate and its oxidation to sulfide, supporting previous observations of S^0^AB growth (1). It also contains a cupredoxin superfamily gene (sulfocyanin-like) clustered with cytochromes that have been shown to be involved in Fe^2+^ oxidation in Sulfobacillus thermosulfidooxidans, which may provide a favorable redox potential for chalcopyrite bioleaching (12). The genome is also predicted to have *feoE*, which is important for survival during anaerobic iron respiration (13), and a natural resistance-associated macrophage protein (NRAMP) family of proteins, which control metal ion transport and homeostasis (e.g., Mn^2+^, Cu^2+^, Fe^2+^, and Co^2+^) and are involved in cellular responses to fluctuating environmental concentrations of metal ions (14). The genome is predicted to contain a suite of genes encoding proteins for sporulation, including *spoIIR* and *spoIIGA*, and genes for acid resistance, such as the voltage-gated potassium channel *kch* and the high-affinity ATP-driven potassium transporter *kdpABCDE*, which are involved in generating a reverse membrane potential that repels the intrusion of protons in acidic environments (15).

### Data availability.

The draft whole-genome shotgun project for *Firmicutes* strain S^0^AB has been deposited in DDBJ/ENA/GenBank under the accession number JALBUF000000000. The version described in this paper is version JALBUF010000000. The raw read data are available under the SRA accession numbers SRX15640773 and SRX15640772. Project data are available under the BioProject accession number PRJNA813843.

## References

[1] Pakostova E, Johnson DB. 2019. Microbial generation of sulfuric acid from granular elemental sulfur in laboratory-scale bioreactors. Hydrometallurgy 190:105152. doi:10.1016/j.hydromet.2019.105152.

[2] Hallberg KB, Johnson DB. 2007. Isolation, enumeration, growth, and preservation of acidophilic prokaryotes, p 1155–1165. *In* Hurst CJ, Crawford RL, Garland JL, Lipson DA, Mills AL, Stetzenbach LD (ed), Manual of environmental microbiology. American Society for Microbiology, Washington, DC.

[3] Ñancucheo I, Oliveira R, Dall'Agnol H, Johnson DB, Grail B, Holanda R, Nunes GL, Cuadros-Orellana S, Oliveira G. 2016. Draft genome sequence of a novel acidophilic iron-oxidizing *Firmicutes* species, “*Acidibacillus ferrooxidans*” (SLC66^T^). Genome Announc 4:e00383-16. doi:10.1128/genomeA.00383-16. 27198020 PMC4888994

[4] Bolger AM, Lohse M, Usadel B. 2014. Trimmomatic: a flexible trimmer for Illumina sequence data. Bioinformatics 30:2114–2120. doi:10.1093/bioinformatics/btu170. 24695404 PMC4103590

[5] Li H. 2011. A statistical framework for SNP calling, mutation discovery, association mapping and population genetical parameter estimation from sequencing data. Bioinformatics 27:2987–2993. doi:10.1093/bioinformatics/btr509. 21903627 PMC3198575

[6] Li H, Handsaker B, Wysoker A, Fennell T, Ruan J, Homer N, Marth G, Abecasis G, Durbin R, 1000 Genome Project Data Processing Subgroup. 2009. The Sequence Alignment/Map format and SAMtools. Bioinformatics 25:2078–2079. doi:10.1093/bioinformatics/btp352. 19505943 PMC2723002

[7] Quinlan AR, Hall IM. 2010. BEDTools: a flexible suite of utilities for comparing genomic features. Bioinformatics 26:841–842. doi:10.1093/bioinformatics/btq033. 20110278 PMC2832824

[8] Wick RR, Judd LM, Gorrie CL, Holt KE. 2017. Unicycler: resolving bacterial genome assemblies from short and long sequencing reads. PLoS Comput Biol 13:e1005595. doi:10.1371/journal.pcbi.1005595. 28594827 PMC5481147

[9] Gurevich A, Saveliev V, Vyahhi N, Tesler G. 2013. QUAST: quality assessment tool for genome assemblies. Bioinformatics 29:1072–1075. doi:10.1093/bioinformatics/btt086. 23422339 PMC3624806

[10] Chun J, Oren A, Ventosa A, Christensen H, Arahal DR, da Costa MS, Rooney AP, Yi H, Xu X-W, De Meyer S, Trujillo ME. 2018. Proposed minimal standards for the use of genome data for the taxonomy of prokaryotes. Int J Syst Evol Microbiol 68:461–466. doi:10.1099/ijsem.0.002516. 29292687

[11] Seemann T. 2014. Prokka: rapid prokaryotic genome annotation. Bioinformatics 30:2068–2069. doi:10.1093/bioinformatics/btu153. 24642063

[12] Christel S, Herold M, Bellenberg S, Buetti-Dinh A, El Hajjami M, Pivkin IV, Sand W, Wilmes P, Poetsch A, Vera M, Dopson M. 2018. Weak iron oxidation by *Sulfobacillus thermosulfidooxidans* maintains a favorable redox potential for chalcopyrite bioleaching. Front Microbiol 9:3059. doi:10.3389/fmicb.2018.03059. 30631311 PMC6315122

[13] Bennett BD, Brutinel ED, Gralnick JA, Löffler FE. 2015. A ferrous iron exporter mediates iron resistance in *Shewanella oneidensis* MR-1. Appl Environ Microbiol 81:7938–7944. doi:10.1128/AEM.02835-15. 26341213 PMC4616933

[14] Nevo Y, Nelson N. 2006. The NRAMP family of metal-ion transporters. Biochim Biophys Acta 1763:609–620. doi:10.1016/j.bbamcr.2006.05.007. 16908340

[15] Vergara E, Neira G, González C, Cortez D, Dopson M, Holmes DS. 2020. Evolution of predicted acid resistance mechanisms in the extremely acidophilic *Leptospirillum* genus. Genes 11:389. doi:10.3390/genes11040389. PMC7231039 32260256

